# Association of Short Tandem Repeat Polymorphism in the Promoter of *Prostate Cancer Antigen 3* Gene with the Risk of Prostate Cancer

**DOI:** 10.1371/journal.pone.0020378

**Published:** 2011-05-31

**Authors:** Wu Zhou, Zhanguo Chen, Wangqiang Hu, Mo Shen, Xiaoxia Zhang, Chengdi Li, Zhiliang Wen, Xiuling Wu, Yuanping Hu, Xiaohua Zhang, Xiuzhi Duan, Xiucui Han, Zhihua Tao

**Affiliations:** 1 Department of Medical Laboratory, The First Affiliated Hospital of Wenzhou Medical College, Zhejiang, People's Republic of China; 2 Department of Medical Laboratory, Ningbo Municipal Hospital of Traditional Chinese Medicine, Zhejiang, People's Republic of China; 3 Department of Urology, The First Affiliated Hospital of Wenzhou Medical College, Zhejiang, People's Republic of China; 4 Department of Pathology, The First Affiliated Hospital of Wenzhou Medical College, Zhejiang, People's Republic of China; 5 Department of B-Ultrasound, The First Affiliated Hospital of Wenzhou Medical College, Zhejiang, People's Republic of China; 6 Department of Tumor Surgery, The First Affiliated Hospital of Wenzhou Medical College, Zhejiang, People's Republic of China; Clermont Université, France

## Abstract

**Background:**

*PCA3 (prostate cancer antigen 3*) gene is one of the most prostate cancer-specific genes at present. Consequently, the prostate-specific expression and the sharp up-regulation of *PCA3* mRNA in prostate cancer suggest a unique transcriptional regulation, which possibly can be attributed to promoter polymorphism. In our study, we evaluated whether there is polymorphism in *PCA3* promoter region and also assess the association of the polymorphism with prostate cancer.

**Methodology/Principal Findings:**

We designed a specific primer set to screen the promoter of *PCA3* gene by polymerase chain reaction (PCR)-based cloning and sequencing with the DNA extracted from peripheral blood samples of prostate cancer (PCa) cases (n = 186) and healthy control cases (n = 135). Genotype-specific risks were estimated as odds ratios (ORs) with associated 95% confidence intervals (CIs) by chi-square test. Possible deviation of the genotype frequencies from controls and PCa cases expected under Hardy-Weinberg equilibrium was assessed by the chi-square test. Short tandem repeat polymorphism of *TAAA* was found in the promoter region of *PCA3* gene, five polymorphisms and eight genotypes were identified. The eight genotypes were divided into three groups: ≤10*TAAA*, 11*TAAA*, ≥12*TAAA*. The group 11*TAAA* and ≥12*TAAA* were associated with higher relative risk for prostate cancer than group ≤10*TAAA* (OR = 1.76, 95%CI = 1.07–2.89[for group 11TAAA]; OR = 5.28, 95%CI = 1.76–15.89[for group ≥12*TAAA*]).

**Conclusions/Significance:**

The presence of the (*TAAA*)n short tandem repeat polymorphisms in the *PCA3* promoter region may be a risk factor for prostate cancer in the Chinese population.

## Introduction

Prostate cancer is the most commonly diagnosed malignancy and the second leading cause of cancer-related deaths in the Western male population [1], and its incidence is still increasing. PCa can be cured by radical surgery or radiation therapy if the disease is localized within the prostate[2]–[5]. However, when this carcinoma has spread locally or distantly, no curative therapy can be offered, and these patients will suffer from a poor prognosis [6], [7] **.** Therefore, there is an urgent need for early diagnosis of the disease to increase the cure rate for PCa.

Although serum prostate-specific antigen (PSA) measurement is regarded as the best conventional serum tumor marker available, there is not enough specificity and sensitivity for PSA in detecting prostate cancer early. For example, PSA is often elevated in benign prostatic hyperplasia and prostatitis. Further, the Prostate Cancer Prevention Trial showed that even in patients with PSA levels <4 ng/ml, >15% had biopsy detectable prostate cancer[8]. More prostate cancer specific and sensitive biomarkers are urgently needed. Recently, Bussemakers *et al*. reported a novel prostate-specific gene, prostate cancer antigen 3 gene, also called *DD3* (differential display code 3) which was found to be 10–100 fold over-expressed in 53 of 56 human prostate cancer samples in a Northern blot analysis whereas it was not expressed in adjacent non-malignant prostatic tissues[9] **.** Furthermore, reverse transcription-PCR analysis using *PCA3*-specific primers indicated that *PCA3* transcripts were not found in a wide range of normal human tissues and other human malignant tumors, *PCA3* is regarded as one of the most prostate cancer-specific genes described so far.

The current understanding is that the *PCA3* gene contains a high density of stop-codons and expresses a non-coding messenger RNA in epithelial prostate cells, it functions as a polyadenylated RNA transcript, but no cytoplasmic protein results from its transcription. The prostate-specific expression and the sharp up-regulation of *PCA3* mRNA in prostate cancer suggest a unique transcriptional regulation. As a result, the *PCA3* gene promoter attracted our attention.

We designed a specific primer set to screen the promoter of *PCA3* gene by PCR -based cloning and sequencing with the DNA extracted from peripheral blood samples of prostate cancer patients and healthy control individuals. We surprisedly found that there were short tandem repeat (STR) polymorphisms in the promoter region of *PCA3* gene and the *TAAA* STR polymorphisms are significant difference between prostate cancer patients and healthy control individuals.

## Results

A total of 321 subjects, including 186 patients with PCa and 135 healthy individuals as control group from The First Affiliated Hospital of Wenzhou Medical College, were analyzed for polymorphisms in promoter of the *PCA3* gene. DNA samples from the blood from individual patients each exhibited a single band after PCR amplification. The cloning and sequencing assay revealed a STR in the promoter region of the *PCA3* gene. Then the sequence of the promoter region of the *PCA3* gene was initially screened for polymorphisms by cloning and sequencing assay in 186 patients with PCa (mean age at diagnosis: 72.33 years; SD: 9.30) and 135 controls (mean age at diagnosis: 71.19 years; SD: 7.39, Table 1). Age at diagnosis did not differ significantly between the patients and the controls (t = 1.20; P = 0.23).

**Table 1:** Clinical characteristics.

Characteristics	Cases	Controls
Subjects(n)	186	135
Age^a^ (yr), mean ± SD	72.33±9.30	71.39±7.39
Gleason score,n (%)		
≤6	33(17.74)	—
7	52(27.96)	—
≥8	101(54.30)	—
PSA ng/ml, Medians (Q25%–Q75%)	34.60 (7.75–100.00)	0.73(0.44–1.33)

a Independent-Samples T Test between cases and controls, t = 1.20, P = 0.23.

**Figure 1:**
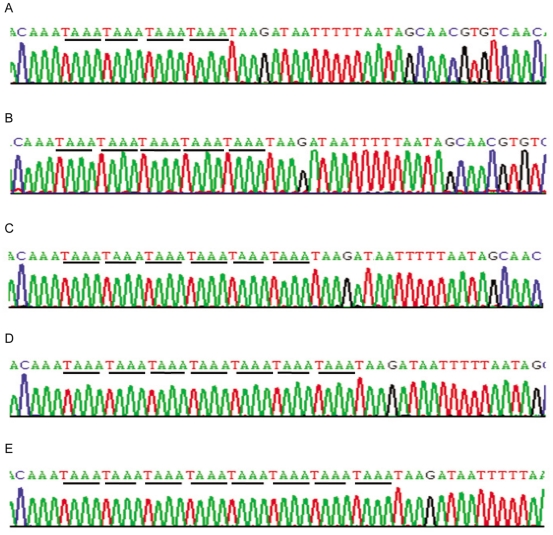
Repressentative PCR -based cloning and sequencing of STR polymorphism in the promoter region of *PCA3* gene. **A**. (*TAAA*)_4_ alleles; **B**. (*TAAA*)_5_ alleles; **C**. (*TAAA*)_6_ alleles; **D**. (*TAAA*)_7_ alleles; **E**. (*TAAA*)_8_ alleles.

In the present study, five polymorphisms were identified: 4, 5, 6, 7, 8 (the number represents the repeat times of *TAAA* in the promoter of *PCA3* gene, Fig 1), and eight genotypes were founded. The 4/5, 4/6, 5/5, 5/6, 5/7, 5/8, 6/6, 6/7 genotypes were observed in 0.5%, 0.5%, 52.7%, 34.4%, 3.3%, 1.6%, 5.4% and 1.6% of PCa patients, respectively. Whereas only the 5/5, 5/6, 6/6genotypes were observed in 71.1%, 25.8%, 3.1% of control subjects, respectively. There was no evidence that the genotype frequencies deviated from those expected under the Hardy-Weinberg equilibrium for patients (χ^2^ = 1.44, df = 3, P = 0.70) and control subjects (χ^2^ = 0.14, df = 1, P = 0.71).

We hypothesized that one *TAAA* was one unit of the transcriptional initiation site of *PCA3* gene, and *TAAA* repeats up-regulated *PCA3* transcription, then the same repeat numbers of *TAAA* might be associated with the same transcription rate and the more *TAAA* repeats, the more *PCA3* gene expression. So based on total number of *TAAA* repeats in the alleles, eight genotypes were divided into three groups: ≤10*TAAA*, 11*TAAA*, ≥12*TAAA* (e.g. the total *TAAA* repeat number of genotype (*TAAA*)n/(*TAAA*)m is x[x = n+m],and belongs to the “x” *TAAA* group).

Chi-square test showed statistical significance difference in the construction of PCa and controls between the three groups (χ^2^ = 13.27, df = 2, P = 0.01), a significant association was observed between the pooled genotypes and PCa risk. The 11*TAAA* carrier was associated with an increased risk for prostate cancer than individual having ≤10*TAAA* (OR = 1.76; 95% CI = 1.07 – 2.89), and the ≥12*TAAA* carrier was also associated with increased risk for prostate cancer than individual having ≤10*TAAA* (OR = 5.28; 95% CI = 1.76–15.89, Table 2).

**Table 2:** Association between *PCA3* promoter STR polymorphisms and prostate carcinoma risk.

Groups	Genotypes	Cases(n)/controls(n)	OR	95%CI	P-value
≤10T*AAA* a, b	4/5,4/6,5/5 c	100/96	1	Ref	
11T*AAA* a	5/6 c	64/35	1.76	1.07–2.89	0.026
≥12T*AAA* a	5/7,5/8,6/6,6/7 c	22/4	5.28	1.76–15.89	0.001

a 10, 11, 12 and 13 correspond to the total number of *TAAA* repeat in one allele.

b 10*TAAA* group includes the 9*TAAA* group.

c 4, 5, 6, 7 and 8 correspond to the number of *TAAA* repeat.

Allele (*TAAA*) _8_ was comparatively rare allele, accordingly, which leaded the samples in 13*TAAA* group were rare, study in larger populations may or may not reveal significant associations with PCa. These observations suggest that additional work is needed to determine the functional significance of this region and the influence of the variability in length and number of *TAAA* repeat in future studies with larger patient cohorts.

There was no association between PCA3 promoter STR polymorphisms and Gleason score in prostate carcinoma patients (r = 0.07, P = 0.342, Table 3).

**Table 3:** Association between *PCA3* promoter STR polymorphism and Gleason score in prostate carcinoma.

Groups	Gleason score			r^e^	P-value
	≤6	7	≥8		
≤10*TAAA*	21	27	52		
11*TAAA*	8	20	36	0.070	0.342
12*TAAA*	3	5	8		
13*TAAA*	1	0	5		

e Spearman's rank correlation coefficient.

## Discussion

Serial analysis of gene expression has shown that *PCA3* expression is significantly and especially up-regulated in the majority of prostatic adenocarcinomas. Therefore, study of genetic alterations in *PCA3* gene may be helpful in elucidating the pathogenesis of prostate cancer.

Gerald W. Verhaegh et al. previous study has shown that no known initiator motif, no *TATA-box*, no *CAAT-box*, and no GC-rich regions were found at consensus positions within the *PCA3* promoter. Therefore, novel and tissue-specific cis-acting elements and trans-acting transcription factors might define the specific and characteristic expression of *PCA3* gene. In their study, a footprint was found further upstream at position −173 to −201 in the *PCA3* promoter. Mutation of this A+T-rich region, resulted in a decreased transcription rate, suggesting a positive role for this element in *PCA3* transcription[10]. In the present study, we surprisedly found that the A+T-rich region within the *PCA3* promoter contained a highly polymorphic region, a STR polymorphism of (*TAAA*)n. STRs are repeating DNA sequences that contain 2 to 6 base-pair units and widespread throughout the human genome and polymorphic in nature. STRs are important genetic markers for mapping studies, disease diagnosis and forensic studies. By screening the A+T-rich region sequence of the promoter, we identified eight STR polymorphisms and found the association between those polymorphisms and PCa risk in Chinese population.

Our results suggested that incidence of PCa was closely related to the repeat numbers of TAAA in the promoter of PCA3, more TAAA repeats associated with increased risk for prostate cancer. In the Chinese population, individuals carrying total 11 repeat numbers of *TAAA* in alleles (group 11*TAAA*) had a 1.76 higher risk of PCa (95% CI = 1.07 – 2.89) than those carrying total ≤10 repeat numbers of *TAAA* in alleles (group ≤10*TAAA*). The group ≥12*TAAA* was also associated with increased risk of prostate cancer than group ≤10*TAAA* (Table 2).

The difference of susceptibility may be related to the difference in allelic frequencies of *PCA3* STR polymorphisms between PCa patients and controls. It is well known that the expression of *PCA3* is very high in the PCa patients than that in healthy individuals. Owing to the *PCA3* promoter has no *TATA-box*, we hypothesized that the transcription would begin from other positions. Instead of *TATA-box*, the (*TAAA*)n polymorphic region may be one of the transcriptional initiation positions. Therefore, the increase of (*TAAA*)n repeats in the promoter of *PCA3* would increase the transcriptional initiation sites of *PCA3* and up-regulated the expression of *PCA3* mRNA in PCa patients. This was in concordance with our earlier published data [11], [12] and the results of Klecka, J. et al. [9], [13]–[15]. These results revealed that the polymorphism of *TAAA* STR in the promoter of *PCA3* maybe is a genetic risk marker for PCa in Chinese population.Of course, this meaningful conclusions of the reapet times of the *TAAA* may modulate *PCA3* expression still need the confirmation of correlation research from different genetic polymorphism types and the *PCA3* mRNA expression in individual or prospective correlation research.

Our study has a number of important limitations, the most important of which is its small sample size,so additional larger studies are needed to provide further insight into these preliminary findings. It is also important to note that this is a prospective study carried out in a regional university hospital over five years and cases of PCa patients are not very frequent. Since the Chinese population generally is genetically more homogeneous than other ethnic populations, we predict similar findings in larger sample sizes across China, but the applicability of our findings to other ethnic populations will need further investigation in varying patient populations before these data can be generally extrapolated to other ethnicities.

In conclusion, our study demonstrates a significant association between *PCA3* promoter STR genetic polymorphisms and PCa in Chinese populations. These findings indicate that the *PCA3* promoter STRs are genetic susceptibility factor for PCa and the STRs might play a role in the development of this cancer, what will give clues for a pilot study of carcinogenesis and development of PCa.

## Materials and Methods

### Ethics statement

This study was approved by the ethics committee of The First Affiliated Hospital of Wenzhou Medical College, China, and was in accordance with the Helsinki declaration. Written informed consent was obtained from each of the participants at the time of enrollment.

### Study design and patients

For this study, 186 patients with prostate cancer that underwent surgery in the Department for Surgery at The First Affiliated Hospital of Wenzhou Medical College between August 2004 and September 2009 were chosen retrospectively. PCa patients wre confirmed by histopathological evaluation, each tumor was graded using the Gleason scoring system by two pathology physicians. In addition, 135 healthy control individuals were included to determine the distribution of polymorphisms in a normal population. The controls were selected from men invited to a systematic health screening, under the proposition of the national health insurance, performed in the same geographical areas than the hospitals where the cases were collected. They were checked by digital rectal examination (DRE), their PSA value had to be less than 4 ng/ml, and they could exhibit no signs of PCa. Unfortunately, we do not know that a man with a normal DRE and a normal PSA will not develop PCa in the future. Statistically, these observations bias the study against finding a significant difference between the cases and controls, since some controls will have prostate carcinoma diagnosed in the future. While male samples were representative of the general clinical population, we cannot exclude unreported histories of prostate disease among the control group. However, such histories should be no more frequent than in a random, non-urologic clinic population.

### DNA extraction

Five milliliters of venous blood were collected in EDTA as a source of peripheral blood leukocytes. Genomic DNA was extracted and purified according to established protocols by using the QIAamp DNA Mini Kit (Qiagen, Hilden, Germany).

### Sequencing and analysis of polymorphisms

DNA was extracted from 500 µL of archived whole blood using the QIAamp DNA Mini Kit (Qiagen, Hilden, Germany). The *PCA3* promoter was amplified by PCR on the basis of the sequence of the *PCA3* gene presented in GenBank accession number AL359314.14 and AF279290.1 with Taq polymerase (Qiagen, Hilden, Germany) with the following primers: 5′- GAT GGG AAC TCA CAT TTG G -3′ (forward) and 5′- CTG ATG CCA GCT TCT CG - 3′ (reverse). PCRs were performed in 50 µL reaction mix containing 1 µL of extracted DNA; 5 µL 10ΧPCR Buffer (100 mmol/L Tris-HCL (pH 9.2)); 3 µL MgCl2(25 mmol/L); 5 µL dNTP (2 mmol/L); 2 µL forward and reverse primer (5 µmol/L), respectively; 0.4 µL Taq-polymerase; and 31.6 µL distilled water. PCR conditions were as follows: 95°C for 4 minutes and then 40 cycles of 95°C for 1 minute, 60°C for 30 seconds, 72°C for 1 minute, and followed by extension at 72°C for 10 minutes. All PCR products were electrophoresed on a 1.5% agarose gel. Amplified fragments were purified by QIAquick Spin PCR Purification Kit (Qiagen, Hilden, Germany),then were sent to Invitrogen Corporation in Shanghai for cloning and sequencing assay with no information on the specimens and knowledge of the study.

### Statistical Analysis

We used the Statistical Package for Social Sciences for Windows (version 13.0; SPSS Inc, Chicago, IL, USA) for statistical analysis. The values for age are reported as mean±SD. Statistical analysis of age was performed by the unpaired t-test. A chi-square test was applied for cross-table analysis. Genotype-specific risks were estimated as odds ratios (ORs) with associated 95% confidence intervals (CIs) by chi-square test possible deviation of the genotype frequencies from PCa and controls expected under Hardy-Weinberg equilibrium was assessed by the chi-square test. Spearman's rank correlation tests was used to assess whether there is a relationship between PCA3 promoter STR polymorphism and Gleason score in PCa. Significance statements refer to P values of 2-tailed tests that were less than 0.05.
